# Physical Activity Programming and Physical Function of Older Adults in Adult Day Centers: A Mixed-Methods Approach

**DOI:** 10.1093/geroni/igaa057.962

**Published:** 2020-12-16

**Authors:** Yuliana Soto, Susan Aguinaga, Jacqueline Guzman

**Affiliations:** 1 University of Illinois--Urbana-Champaign, champaign, Illinois, United States; 2 University of Illinois at Urbana-Champaign, Urbana, Illinois, United States

## Abstract

With increased prevalence of Alzheimer’s disease, there is a need for long-term care services (e.g., Adult Day Centers (ADCs)) to provide physical activity (PA) programs to maintain physical function of older adults. ADCs report offering PA programs; however, information on PA programs and physical function of participants attending ADCs is limited. The study aims to a) explore perspectives of ADC directors on PA programming; b) examine physical function in older adults attending ADCs. A cross-sectional mixed-methods study was conducted among ADC directors and attending participants. Interviews were conducted with ADC directors to assess barriers and facilitators of PA programming. Physical function was assessed among ADC participants via the Short Physical Performance Battery (SPPB) and Timed Up and Go (TUG). Five director interviews were conducted and three major themes emerged; 1) current PA programming limited by fear of falls, 2) staff training and retention, and 3) diversifying PA programming. Twenty-nine ADC participants enrolled in the study, Mage= 74.5±8.2 years; BMI= 29.2 ±7.4 kg/m2; MMSE= 25.6 ±3.3; 51.7% (n=15) African American; 79.3% (n=23) males. ADC participants scored 6.7±3.1 on the SPPB and 15.4±5.3 seconds on the TUG. Directors expressed the importance of PA; however, mentioned current programming was limited due to risk of falls and untrained staff in PA. Findings indicate that older adults attending ADCs have physical function scores indicative of high fall risk. Future PA programming may consider including alternative forms of PA while embedding falls prevention strategies to reduce risk of falls and improve physical function among ADC participants.

